# Excess deaths associated with the Iranian COVID-19 epidemic: A province-level analysis

**DOI:** 10.1016/j.ijid.2021.04.015

**Published:** 2021-06

**Authors:** Mahan Ghafari, Alireza Kadivar, Aris Katzourakis

**Affiliations:** a Department of Zoology, University of Oxford, Oxford, UK; b Center for Statistical and Operational Research, Statsminute Company, Tehran, Iran

**Keywords:** COVID-19, SARS-CoV-2, Excess mortality, Infection fatality ratio, Iran

## Abstract

**Objective:**

There has been no province-level data on the number of coronavirus disease 2019 (COVID-19)-related deaths in Iran since the start of the pandemic. This study was performed to estimate the number of COVID-19 deaths and population-level exposure per province using seasonal all-cause mortality data.

**Methods:**

Time-series data were collected from the National Organization for Civil Registration on the seasonal all-cause mortality from spring 2015 to summer 2020 (from March 21, 2015 to September 21, 2020), in accordance with the Solar Hijri (SH) calendar, to estimate the expected number of seasonal deaths for each province using a piecewise linear regression model. A population-weighted infection fatality ratio was then applied to estimate the level of exposure per province during this period.

**Results:**

From the start of winter to the end of summer (from December 22, 2019 to September 21, 2020), there were a total of 58 900 (95% confidence interval 46 900–69 500) excess deaths across all 31 provinces, with 27% (95% confidence interval 20–34%) estimated nationwide exposure to severe acute respiratory syndrome coronavirus 2 (SARS-CoV-2). In particular, Qom and Golestan were among the hardest-hit provinces, with nearly 57% exposure, while another 27 provinces showed significant levels of excess mortality in at least one season with >20% population-level exposure to the virus. Unexpectedly high levels of excess mortality were also detected during fall 2019 (from September 23 to December 21, 2019) across 18 provinces, unrelated and prior to the start of the COVID-19 pandemic.

**Conclusions:**

This study quantified the pattern of spread of COVID-19 across the country and identified areas with the largest epidemic growth requiring the most immediate interventions.

## Highlights

• This is the first province-level study to examine COVID-19-related deaths in Iran.
• There were nearly 59 000 extra deaths during the epidemic until September 21, 2020.
• Some provinces such as Qom and Golestan reached >50% exposure to SARS-CoV-2.
• Eighteen provinces recorded significant levels of excess mortality during the fall of 2019.

## Introduction

Information on all-cause mortality in Iran and its changing pattern over time represents a major source for evaluating the burden of the coronavirus disease 2019 (COVID-19) epidemic across the country. In order to estimate the number of deaths in excess of previous years, i.e., the mortality attributable to a public health crisis, the total number of registered deaths during the time of the crisis can be compared to the ‘background’ level measured during periods with no major crisis (Checchi and Roberts, 2005). Like many other developing countries, Iran has incomplete records of registered deaths. A recent report from 2015 (United Nations Statistics Division, 2015) showed that the estimated coverage of registered births and deaths in Iran was 97% and 92%, respectively. Coverage information was obtained from country representative(s) either from the National Statistical Office or the Civil Registration Authority attending one of the United Nations Statistics Division workshops. The National Organization for Civil Registration of Iran (NOCR) is responsible for the registration of births, marriages, divorces, and deaths across the country. It initially updates the number of registered (crude) deaths at the end of every season for all 31 provinces of Iran (in accordance with the Solar Hijri (SH) calendar date). More detailed data on the number of registered deaths per month then follow at the end of each calendar year (on the March equinox) along with an annual report from the Ministry of Health and Medical Education (MoHME), which includes an aggregate record of causes of death (based on the International Statistical Classification of Diseases and Related Health Problems, 10th revision) according to individual age, sex, and place of residence (Khosravi et al., 2007a).

**Figure 1:**
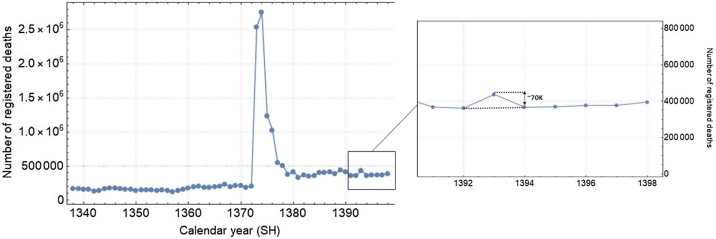
Annual number of registered deaths in Iran over the last 70 years (data from the National Organization for Civil Registration). The inset shows the variation in registered deaths during the more recent years; this appears to have stabilized, with the exception of year 1393 SH, in which there were approximately 70 000 deaths above baseline.

Upon examination of the historical trends in all-cause mortality in Iran from the 1960s to the present, significant levels of variation over time can be observed (Figure 1). In particular, there was a spike in 1995, with more than 2.7 million registered deaths in just one year (a record-high, with a death toll almost 15 times that of previous years). This was the result of an ‘emergency operation’ to record significant levels of under-counting from previous years, such as the casualties of the Iran–Iraq war in the 1980s, and a redesign of NOCR’s data collection strategy (Khosravi et al., 2007a). Between 1966 and 1995, the mortality data based on cemetery records were collected only in a sample of 24 cities. In 1995, the system was redesigned to cover the entire country. Since then, the average number of registered deaths has roughly doubled, with approximately 389 000 deaths compared to 175 000 in previous years. However, even after the completion of the emergency operation (from 1994 to 1999), the annual numbers continued to fluctuate significantly over time, with amplitude variations as large as 100 000 deaths between consecutive years. These variations were so high that even some of the major natural catastrophes in Iran such as the Manjil–Rudbar earthquake with more than 40 000 reported deaths in 1990 and the Bam earthquake with more than 50 000 deaths in 2003 did not have a detectable footprint in the annual all-cause mortality data. Previous works have shown that despite the progress since 1995 in reporting the true number of registered deaths, variations may persist due to substantial delays in the registration and recording of deaths (Khosravi et al., 2007b). Examining the underlying cause(s) of these large variations is beyond the scope of this study, but we note that this is an area that requires further in-depth investigation.

MoHME has not released provincial data on the number of confirmed COVID-19 deaths and only announced the number of cases during the first 33 days following the start of the epidemic (from February 19 to March 22, 2020, with the exception of March 2 and March 3). Thus, seasonal excess mortality is the only proxy to assess the geographical spread of the epidemic across the country. A similar method was used to monitor excess mortality in Africa during a measles outbreak (Rasambainarivo et al., 2020). Despite significant fluctuations in NOCR data over the years, they seem to have remained stable since 1394 SH (Figure 1). Therefore, the present study only incorporated data from the beginning of spring 1394 SH (March 21, 2015) onwards to estimate excess mortality rates (see Methods section).

## Methods

To calculate the expected number of deaths in one season of a particular year for one province, a linear regression model was applied using the reported deaths from that particular season starting from the year 1394 SH until 1 year prior to the year of interest; anything above 2 standard deviations from the mean (95% confidence interval (95% CI)) was considered to be a significant level of excess mortality. The age demographics of each province (Statistical Center of Iran, 2020) were used to calculate the population-weighted infection fatality rate (IFR) (O’Driscoll et al., 2020). For a province with a total population size, *P*, this is given by $\text{I}\text{F}\text{R}=\;\sum_{i}^{M}w_{i}\;\text{I}\text{F}{\text{R}}_{i}$; where *M* is the total number of age groups, $\text{I}\text{F}{\text{R}}_{i}$ is the IFR of age-group *i* with population size $p_{i}$, and $w_{i}=p_{i}/P$. Thus, assuming homogeneous social contact patterns across the population, all deaths attributable to COVID-19 are weighted proportionally among the different age-groups according to their respective population size. To determine the number of individuals exposed to the virus in each province, *Z*, the estimated number of COVID-19-associated deaths based on excess mortality, *X*, was divided by the IFR, *Y*, such that the corresponding variance (Var) in *Z* is given by

$$Var(Z)=Var(X/Y)=E(X^{2})E(1/Y^{2})-E^{2}(X)E^{2}(1/Y)$$

where *X* is a normally distributed number, and the standard deviation of *Y* is given by $\sigma_{Y}=\sqrt{\sum_{i}^{M}w_{i}^{2}\sigma_{i}^{2}}$. The standard deviation of each age group, *i*, was computed using the delta method and is given by $\sigma_{i}=(95\%\;upper\;limit\;-\;95\%\;lower\;limit)\;/\;z_{1-\alpha /2}$, where $z_{1-\alpha /2}$ is the (1-α/2) quantile of the standard normal distribution. Thus, the inverse of *Y* is a random number drawn from the reciprocal normal distribution.

## Results

### Did the COVID-19 epidemic in Iran start during the fall of 2019?

Upon examination of the seasonal trends in mortality during the fall in earlier reports (Ghafari and Madani, 2020, Kadivar, 2020a, Kadivar, 2020b), it was noticed for the first time that there was a spike across the country in 1398 SH (from September 23 to December 21, 2019) when compared to previous years. This was particularly unexpected, as it occurred before the start of the COVID-19 pandemic in December 2019 (Andersen et al., 2020, Lu et al., 2020) and its emergence in Iran during January 2020 (Ghafari et al., 2020a). This led to some speculation about the cryptic transmission of the virus across the country in the fall of 2019.

The study analysis showed an 8% rise in nationwide mortality rate during the fall, with a total of 6040 (95% CI 3480–8600) extra recorded deaths in 18 provinces. This is also aligned with estimates from other studies (Tadbiri et al., 2020). For these extra deaths in the fall to be associated with COVID-19, it would be expected, due to the absence of any non-pharmaceutical interventions at the time, that similar (or higher) levels of excess mortality would be detected in the same provinces during the winter. However, only five provinces were found to have had significantly higher levels of mortality in the winter of 1398 SH (December 22, 2019 to March 19, 2020) (Table 1). These provinces were Qom, Gilan (Guilan), Golestan, Mazandaran, and Qazvin, which were reportedly hit the hardest by an early wave of the COVID-19 epidemic, with a total of 3480 (95% CI 3170–3780) combined extra deaths (Ghafari et al., 2020b). This observation rejects any possible association between the excess mortality in the fall and significant levels of undetected COVID-19-related deaths across the country.

**Table 1:** Seasonal trend of provinces with (+) and without (−) significant levels of excess mortality from the fall of 1398 SH to the summer of 1399 SH.

Provinces	Fall 98	Winter 98	Spring 99	Summer 99
	(Sep 23 to Dec 21, 2019)	(Dec 22, 2019 to Mar 19, 2020)	(Mar 20 to Jun 20, 2020)	(Jun 21 to Sep 21, 2020)
East Azerbaijan	+	−	+	+
West Azerbaijan	+	−	+	+
Ardabil	−	−	+	+
Isfahan	+	−	+	+
Alborz	−	−	+	+
Ilam	+	−	+	+
Bushehr	−	−	−	+
Tehran	+	−	+	+
South Khorasan	−	−	+	−
Razavi Khorasan	−	−	+	+
North Khorasan	+	−	+	+
Khuzestan	−	−	+	+
Zanjan	+	−	+	+
Semnan	+	−	+	+
Sistan and Baluchistan	−	−	−	+
Fars	+	−	−	+
Qazvin	+	+	+	+
Qom	+	+	+	+
Kurdistan	+	−	+	+
Kerman	−	−	+	+
Kermanshah	−	−	+	+
Kohgiluyeh and Boyer-Ahmad	−	−	+	+
Lorestan	+	−	+	+
Mazandaran	+	+	+	+
Markazi	+	−	+	+
Hormozgan	−	−	+	+
Hamedan	−	−	+	+
Yazd	+	−	+	+
Chaharmahal and Bakhtiari^a^	−	−	+	−
Golestan	+	+	+	+
Gilan (Guilan)	+	+	+	+
Nationwide	+	−	+	+

SH, Solar Hijri calendar.

a The number of registered deaths for summer 1394 SH were omitted from the analysis for this province, as this period showed a record-high number with nearly three times more deaths compared to any of the following years.

In search of possible causes of the excess deaths during the fall, the World Health Organization annual influenza situation reports for the Eastern Mediterranean Region in the past 2 years were investigated and a 20% increase in the number of positive flu cases during the fall of 1398 SH was found (with co-circulation of both type A H1N1 and type B influenza in Iran) (Ghafari and Madani, 2020). However, in the absence of surveillance data for influenza-like illnesses or severe acute respiratory infections in Iran, it is not possible to assess the burden of the flu epidemic across the country.

### The geographical spread of COVID-19 in Iran

**Figure 2:**
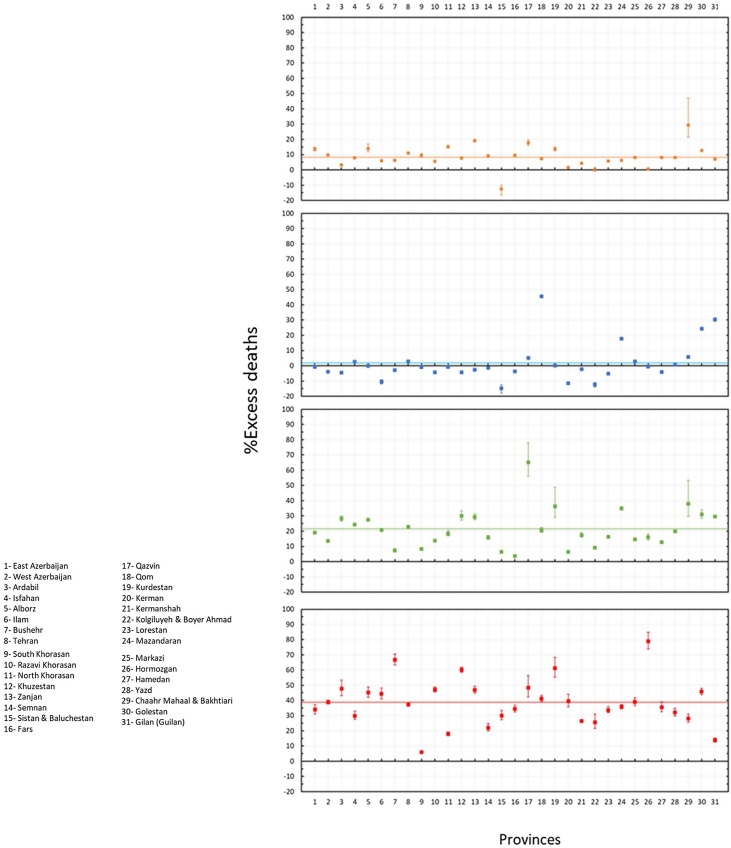
Percentage of excess deaths (compared to the background level) in the fall of 1398 SH (from September 23 to December 21, 2019), winter of 1398 SH (from December 22 to March 19, 2020), spring of 1399 SH (from March 20 to June 20, 2020), and summer of 1399 SH (from June 21 to September 21, 2020). The horizontal solid lines show the mean percentage of excess mortality in the fall (orange), winter (blue), spring (green), and summer (red).

While the mean nationwide excess mortality was only approximately 2% during the winter, it rose to 21% during the spring of 1399 SH (from March 20 to June 20, 2020), with a total of 18 360 (95% CI 13 510–23 210) excess deaths in 28 provinces, and then to 39% during the summer of 1399 SH (from June 21 to September 21, 2020), with a total of 36 280 (95% CI 30 260–42 300) excess deaths in 30 provinces (Figure 2).

**Figure 3:**
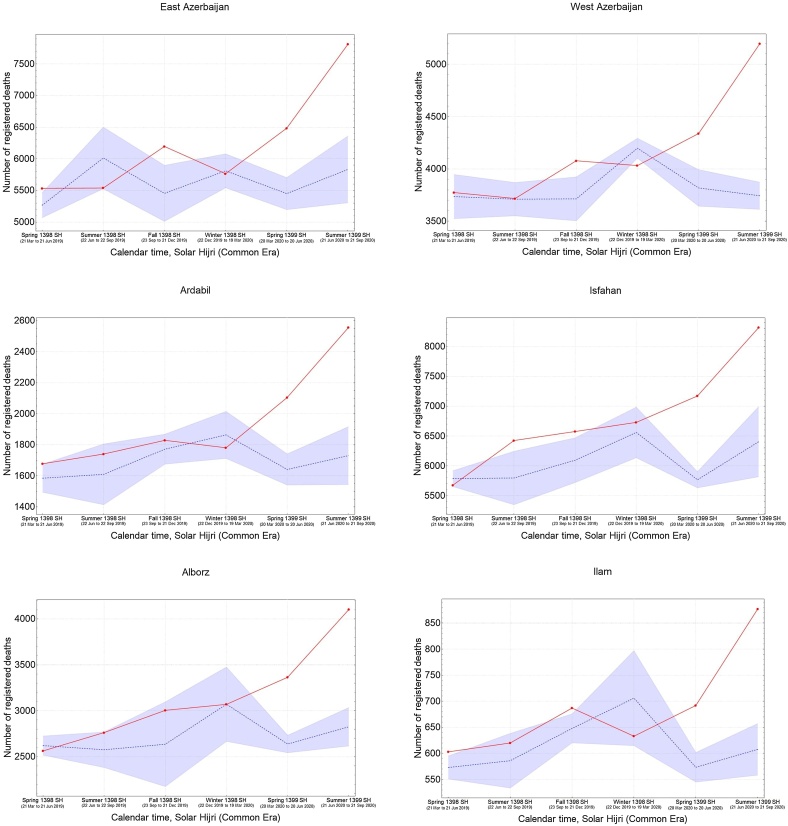

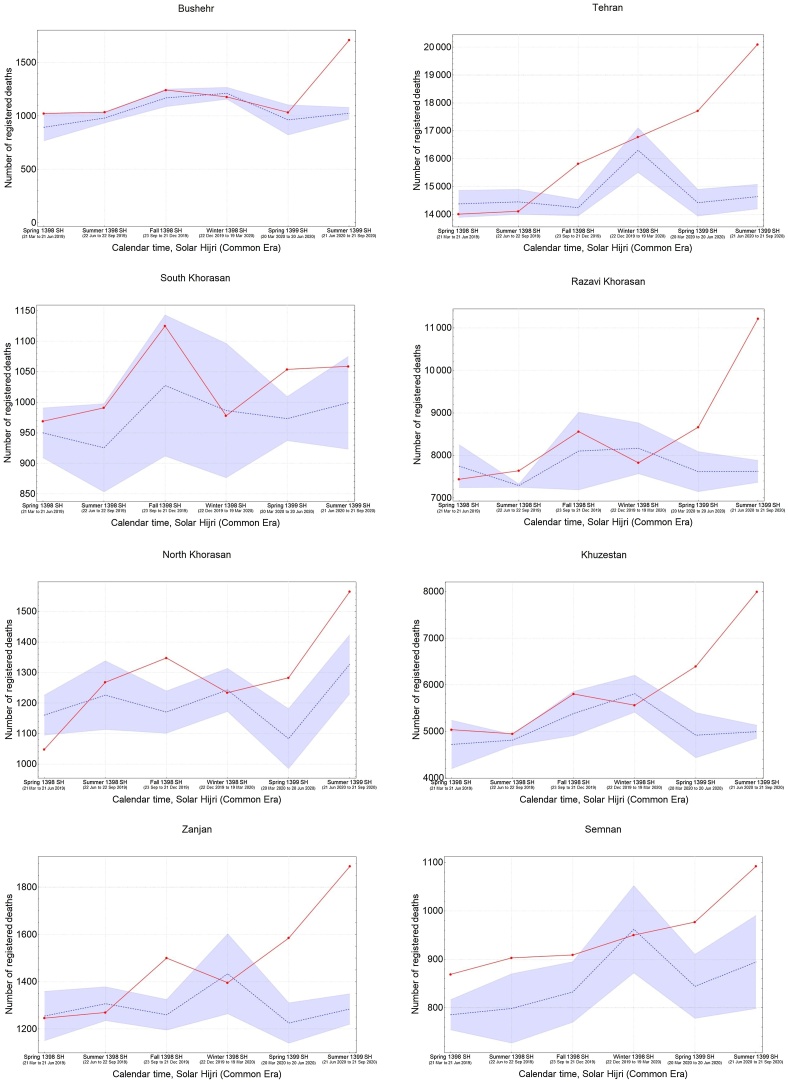

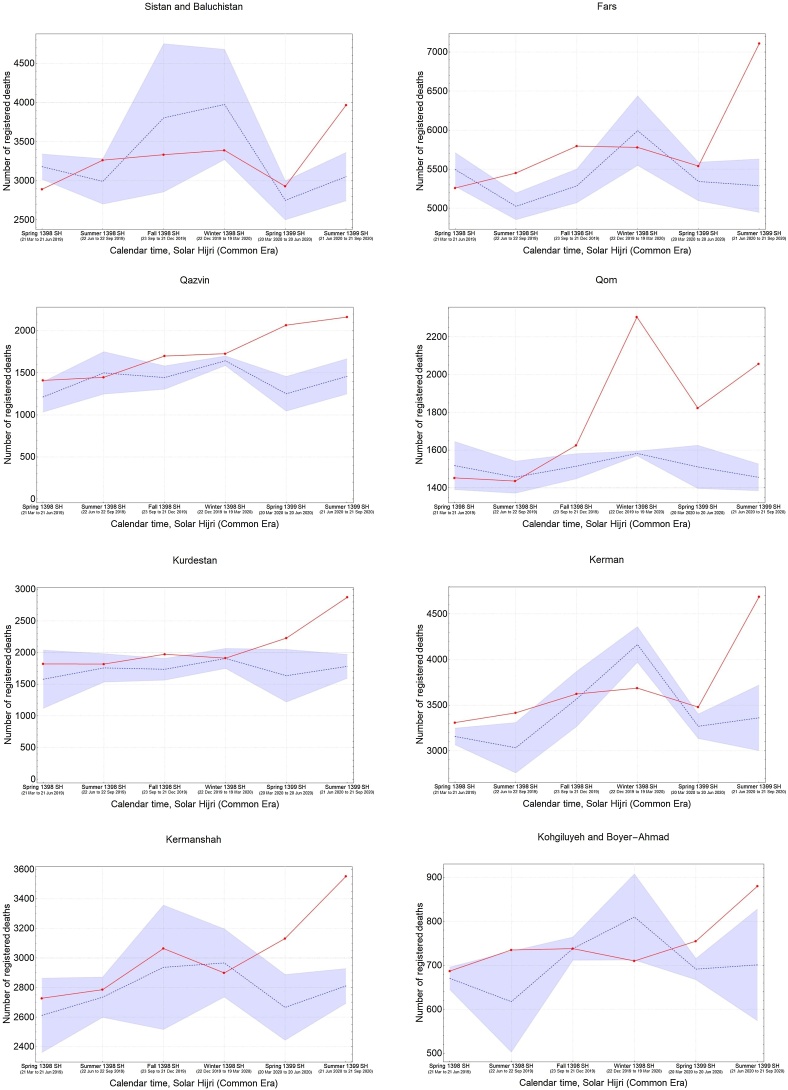

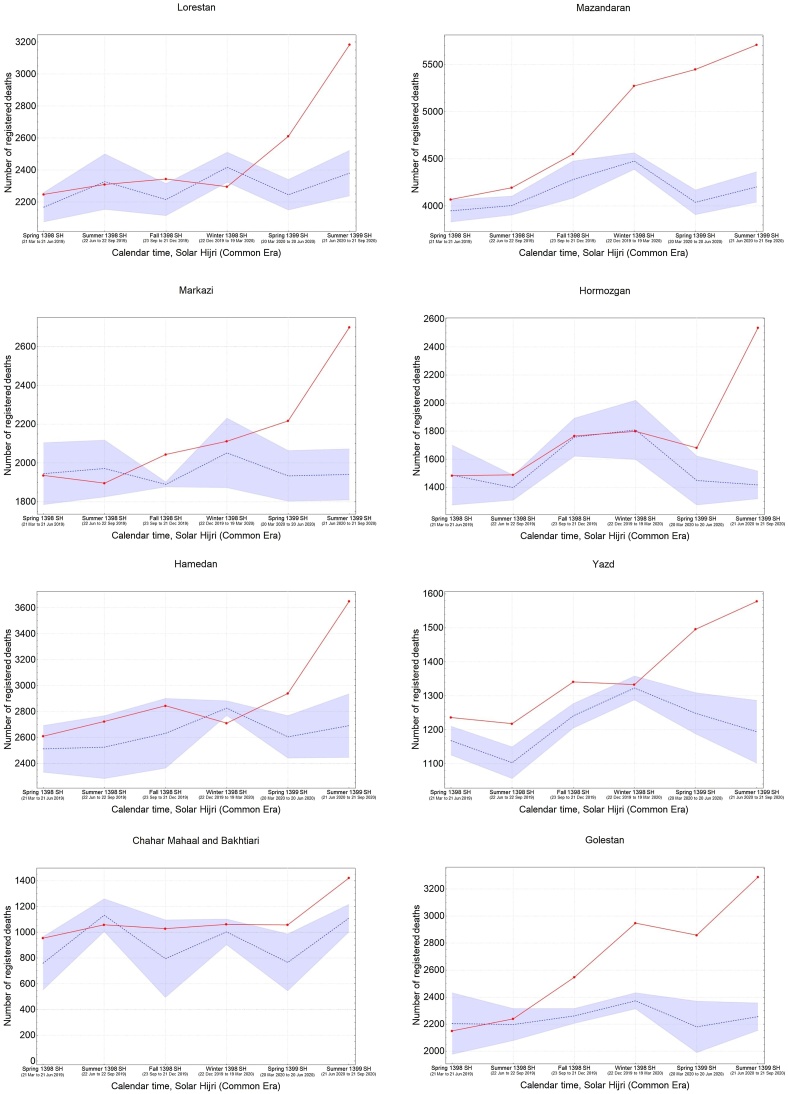

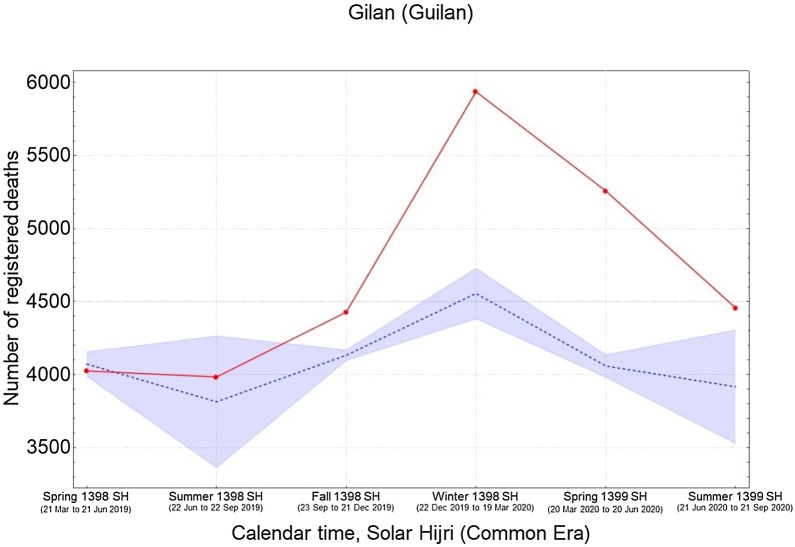
The pattern of excess mortality from spring 1398 SH (March 21, 2019) to summer 1399 SH (September 21, 2020) in each province.

It was also found that the five provinces in the central north part of Iran with an early outbreak of COVID-19 in the winter (i.e., Qom, Gilan, Golestan, Mazandaran, and Qazvin) continued to have significantly high levels of mortality during the spring and summer, while three provinces in the south (Fars, Bushehr, and Sistan-Baluchistan) only showed a rise in mortality during the summer (Figure 3). The remaining provinces had significant levels of excess mortality in the spring, followed by a higher increase during the summer, with the exception of South Khorasan, which only showed significant excess deaths in spring. This indicates that a sustained circulation of the virus had been going on for at least 6 months in most provinces (Figure 3). It was also noted that the level of excess mortality in Gilan continued to decrease after its initial peak during the winter, while Qom seemed to show another increase in the summer after a temporary drop during the spring.

### Estimating the level of exposure across the population

**Figure 4:**
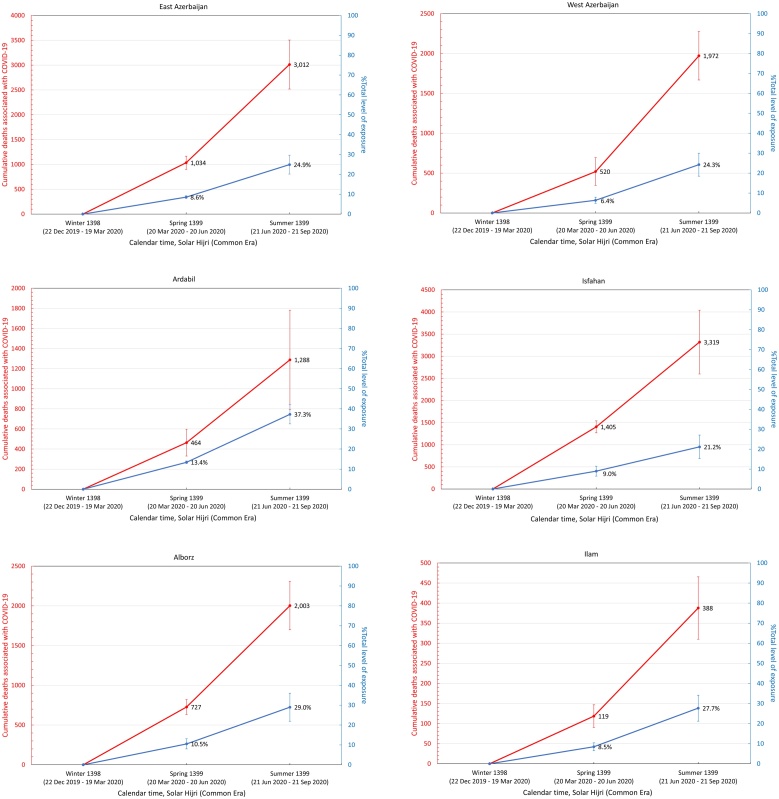

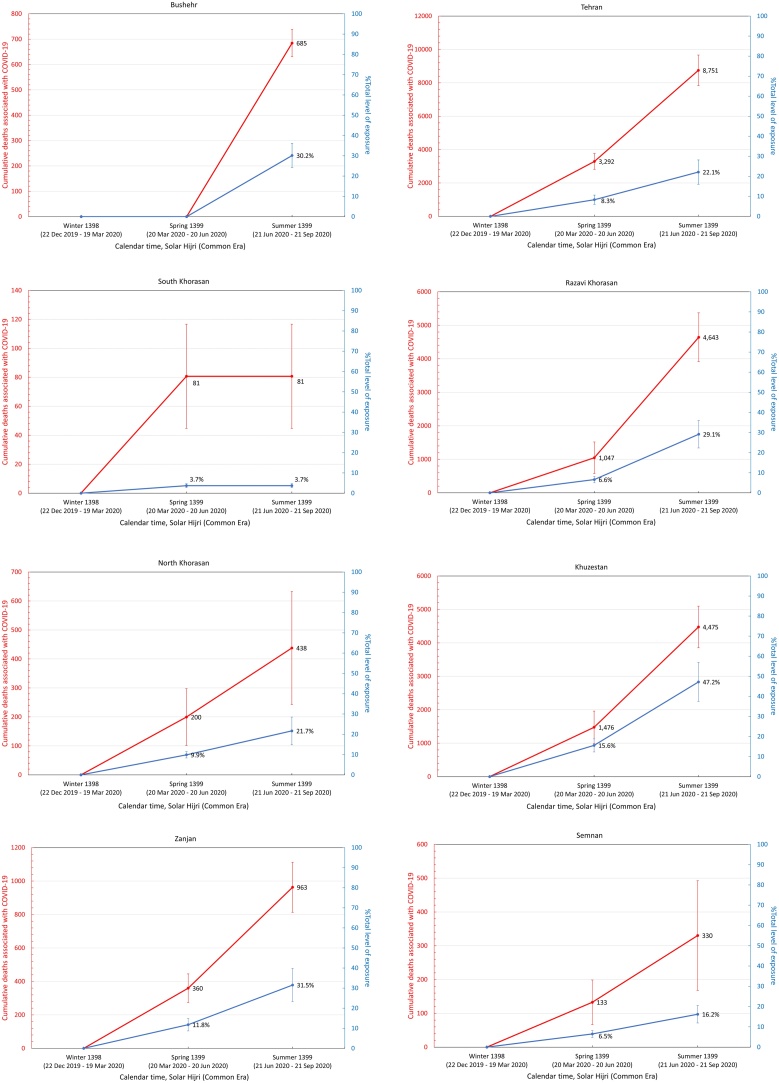

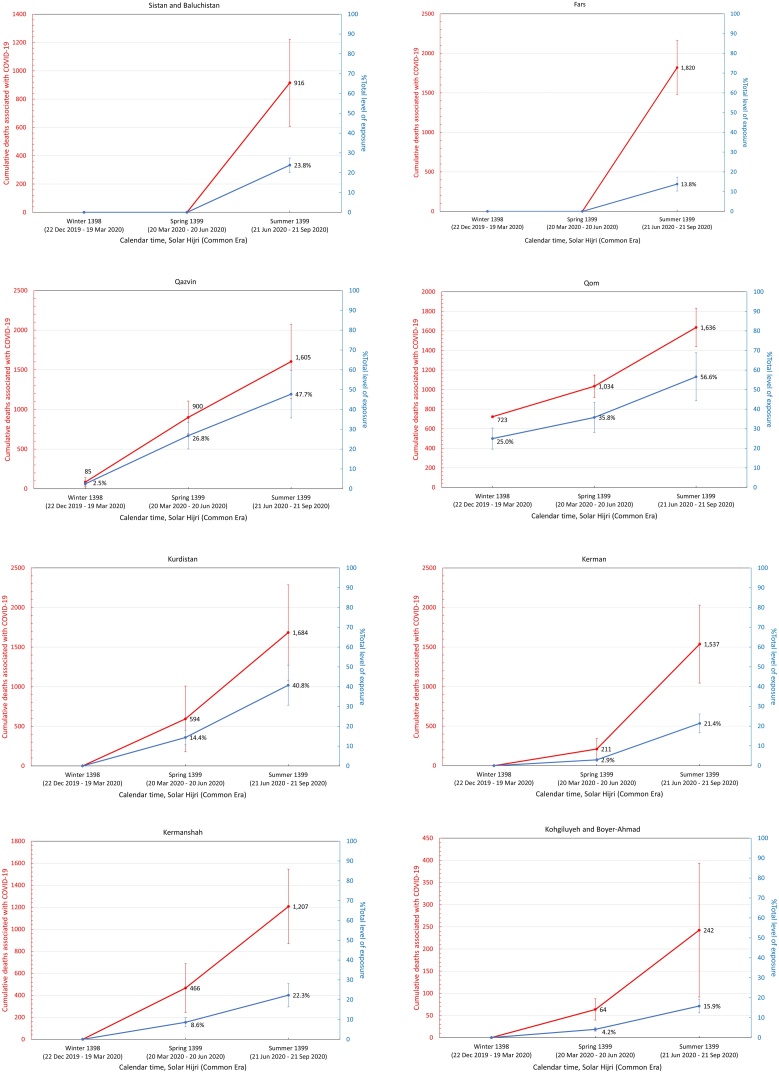

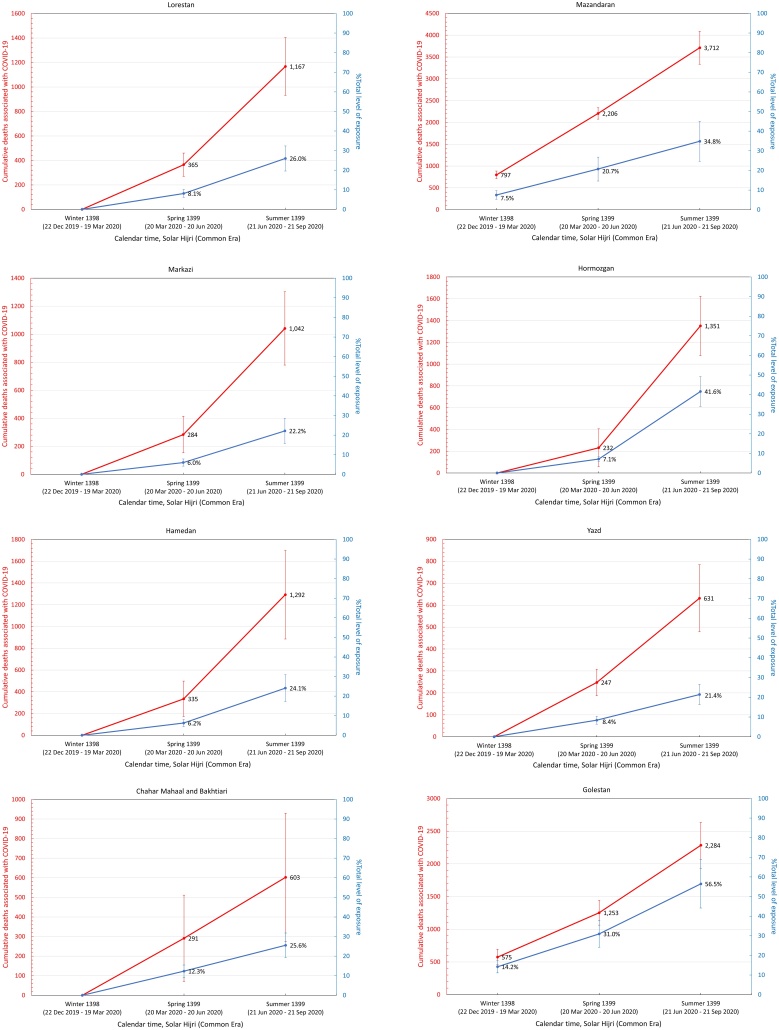

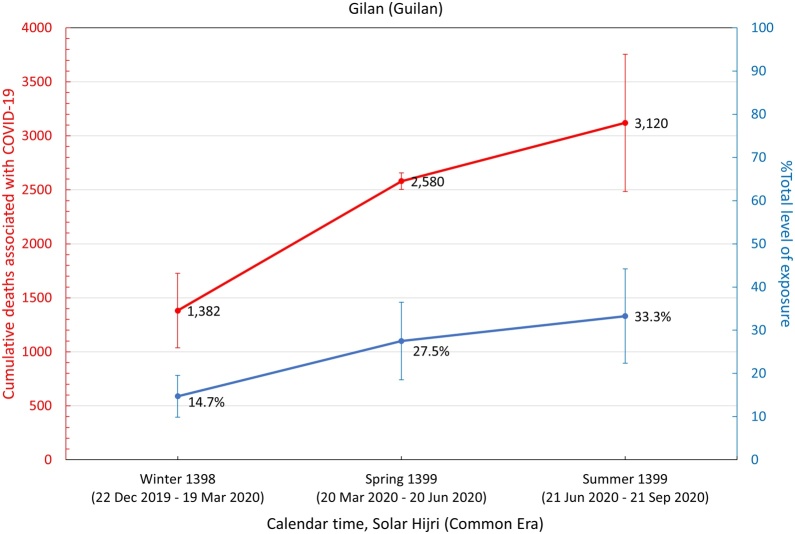
Cumulative number of COVID-19-related deaths (red) and total fraction of individuals exposed to SARS-CoV-2 (blue) in each province from winter 1398 SH to summer 1399 SH.

Assuming that all excess deaths from winter 1398 SH onwards were directly associated with the COVID-19 epidemic, a population-weighted IFR (O’Driscoll et al., 2020) was applied to estimate the level of exposure in each province (see Methods section) by the end of summer 1399 SH (Table 2). It was found that 13 out of 31 provinces had nearly 30% exposure or above, with Qom and Golestan having the highest percentage of individuals exposed at 57% (95% CI 44–69%) and 56% (95% CI 44–69%), respectively (Figure 4).

**Table 2:** Excess mortality associated with COVID-19 and estimated level of exposure in each province. The age structure of each province obtained from the Statistical Centre of Iran was used to calculate the corresponding population-weighted infection fatality rate (IFR). The 95% confidence intervals are shown in parenthesis.

Provinces	Excess deaths	IFR %	Number of individuals exposed	% Exposed
East Azerbaijan	3.0 K (2.2 K – 3.8 K)	0.30 (0.28–0.32)	1.01 M (0.72 M–1.29 M)	25 (18–32)
West Azerbaijan	2.0 K (1.7 K – 2.3 K)	0.24 (0.22–0.25)	0.83 M (0.64 M–1.03 M)	24 (18–30)
Ardabil	1.3 K (1.0 K – 1.6 K)	0.26 (0.25–0.28)	0.49 M (0.36 M–0.61 M)	37 (28–47)
Isfahan	3.3 K (2.6 K – 4.0 K)	0.29 (0.27–0.31)	1.13 M (0.81 M–1.45 M)	21 (15–27)
Alborz	2.0 K (1.7 K – 2.3 K)	0.24 (0.22–0.25)	0.84 M (0.64 M–1.05 M)	29 (22–36)
Ilam	0.4 K (0.3 K – 0.5 K)	0.23 (0.22–0.25)	0.17 M (0.13 M–0.21 M)	28 (21–34)
Bushehr	0.7 K (0.6 K – 0.7 K)	0.18 (0.17–0.19)	0.38 M (0.30 M–0.45 M)	30 (24–36)
Tehran	8.8 K (7.8 K – 9.7 K)	0.28 (0.26–0.30)	3.09 M (2.24 M–3.94 M)	22 (16–28)
South Khorasan	0.1 K (0.0 K – 0.1 K)	0.26 (0.24–0.29)	0.03 M (0.02M–0.04 M)	4 (3–5)
Razavi Khorasan	4.6 K (3.9 K – 5.4 K)	0.23 (0.22–0.25)	2.00 M (1.53 M–2.47 M)	29 (22–36)
North Khorasan	0.4 K (0.2 K – 0.6 K)	0.22 (0.21–0.24)	0.19 M (0.15 M–0.24 M)	22 (17–27)
Khuzestan	4.5 K (3.9 K – 5.1 K)	0.19 (0.28–0.20)	2.33 M (1.85 M–2.81 M)	47 (37–57)
Zanjan	1.0 K (0.8 K – 1.1 K)	0.28 (0.26–0.30)	0.35 M (0.26M–0.44 M)	32 (23–40)
Semnan	0.3 K (0.2 K – 0.5 K)	0.27 (0.25–0.29)	0.12 M (0.09 M–0.16 M)	16 (12–21)
Sistan and Baluchistan	0.9 K (0.6 K – 1.2 K)	0.13 (0.12–0.13)	0.73 M (0.62M–0.84 M)	24 (20–27)
Fars	1.8 K (1.5 K – 2.2 K)	0.26 (0.24–0.28)	0.70 M (0.52M–0.88 M)	14 (10–17)
Qazvin	1.6 K (1.1 K – 2.1 K)	0.25 (0.23–0.27)	0.64 M (0.48M–0.80 M)	48 (36–60)
Qom	1.6 K (1.4 K – 1.8 K)	0.21 (0.19–0.22)	0.79 M (0.62M–0.96 M)	57 (44–69)
Kurdistan	1.7 K (1.1 K – 2.3 K)	0.25 (0.23–0.26)	0.68 M (0.51M–0.85 M)	41 (31–51)
Kerman	1.5 K (1.0 K – 2.0 K)	0.22 (0.20–0.23)	0.72 M (0.56 M–0.87 M)	21 (13–26)
Kermanshah	1.2 K (0.9 K – 1.5 K)	0.27 (0.25–0.29)	0.45 M (0.33 M–0.56 M)	22 (16–28)
Kohgiluyeh and Boyer-Ahmad	0.2 K (0.1 K – 0.4 K)	0.20 (0.19–0.22)	0.12 M (0.09 M–0.15 M)	16 (12–19)
Lorestan	1.2 K (0.9 K – 1.4 K)	0.25 (0.23–0.27)	0.47 M (0.35 M–0.58 M)	26 (20–32)
Mazandaran	3.7 K (3.3 K – 4.1 K)	0.31 (0.29–0.34)	1.18 M (0.83 M–1.53 M)	35 (25–45)
Markazi	1.0 K (0.8 K – 1.3 K)	0.32 (0.29–0.34)	0.33 M (0.23M–0.42 M)	22 (16–29)
Hormozgan	1.4 K (1.1 K – 1.6 K)	0.17 (0.16–0.18)	0.81 M (0.66M–0.96 M)	42 (34–49)
Hamedan	1.3 K (0.9 K – 1.7 K)	0.30 (0.28–0.32)	0.43 M (0.31 M–0.55 M)	24 (17–31)
Yazd	0.6 K (0.5 K – 0.8 K)	0.24 (0.22–0.26)	0.27 M (0.20 M–0.33 M)	21 (16–27)
Chaharmahal and Bakhtiari	0.6 K (0.3 K – 0.9 K)	0.24 (0.22–0.26)	0.25 M (0.19 M–0.31 M)	26 (19–32)
Golestan	2.3 K (1.9 K – 2.6 K)	0.20 (0.19–0.22)	1.12 M (0.87 M–1.36 M)	56 (44–69)
Gilan (Guilan)	3.1 K (2.5 K – 3.8 K)	0.36 (0.34–0.39)	0.86 M (0.57 M–1.14 M)	33 (22–44)
Nationwide	58.9 K (46.9 K – 69.5 K)	0.25 (0.24–0.27)	23.0 M (17.2M–28.7 M)	27 (20–34)

K, thousand; M, million.

**Figure 5:**
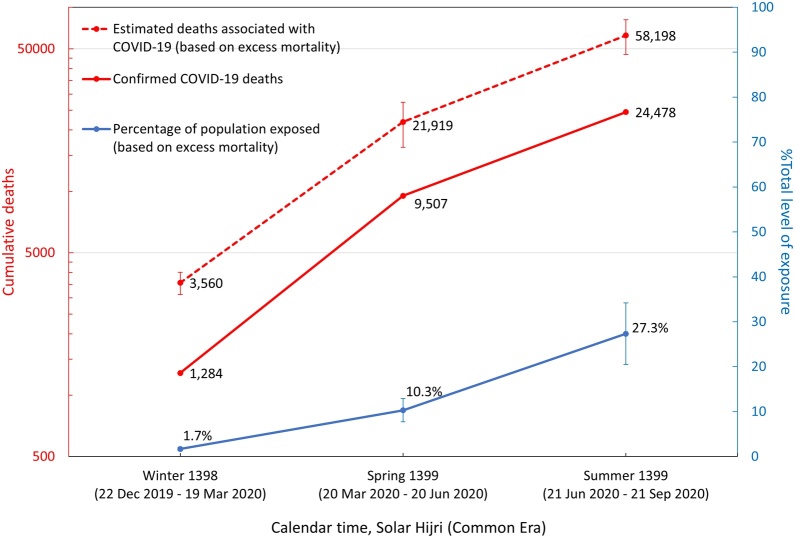
Estimated number of deaths and exposures to COVID-19 based on excess mortality data from winter 1398 SH to summer 1399 SH. Confirmed COVID-19 deaths were collected from the daily reports of the Ministry of Health and Medical Education.

By combining the estimated number of deaths associated with COVID-19 based on excess mortality and the number of individuals exposed to severe acute respiratory syndrome coronavirus 2 (SARS-CoV-2) in each province, the nationwide number of COVID-19-associated infections and deaths can be found (Figure 5). It was calculated that the corrected number of nationwide COVID-19-related deaths during each season was approximately 2.5 times higher than the number of confirmed deaths from the MoHME and that both estimates followed a similar trajectory over time, with particularly elevated levels of excess mortality during the winter (i.e., approximately 2.8 times higher than the confirmed deaths at the time). This may indicate that a larger portion of deaths during the early stages of the epidemic were under-counted, as also noted in our previous analysis (Ghafari et al., 2020b).

## Discussion

In this study, mortality rates in 31 provinces of Iran from fall 1398 SH to summer 1399 SH were compared, with the aim of detecting and measuring excess deaths related to the COVID-19 epidemic. The study findings suggest a total of 58 900 (95% CI 46 900–69 500) COVID-19-related deaths and up to 23.0 million (95% CI 17.2–28.7 million) individuals exposed to SARS-CoV-2 by September 21, 2020, with some of the hardest-hit provinces such as Qom and Golestan reaching up to approximately 57% (95% CI 44–69%) exposure levels.

Given that the epidemic was never fully brought under control, with three major peaks in late-March, mid-July, and late-November that overwhelmed hospitals (as of November 30, 2020), some provinces are experiencing among the highest known population-level prevalence in the world, comparable to Manaus, Brazil (Buss et al., 2020, Hallal et al., 2020). Recent reports from Iranian health officials indicate that up to half of suspected hospitalized cases are not being tested due to shortages of test kits; however, these patients are being treated for COVID-19 based on their clinical symptoms. This highlights the large degree of under-reporting in the actual number of COVID-19 cases and deaths, which also seems to be aligned with our findings that the actual number of COVID-19-related deaths could be up to around 2.5 times higher than the number of confirmed deaths.

The earliest nationwide serology report (National Committee on COVID-19 Epidemiology, 2020) estimated that around 10–15 million Iranians (11.9–17.9% of the population) were infected by late spring. Later in mid-summer, a statement from the then Deputy Minister of Research and Technology of the MoHME was released by the press (Zeinalipour, 2020), reporting that approximately 25 million (29.8% of the population) had so far been exposed to the virus based on recent seroepidemiological analyses. In addition, a detailed antibody seroprevalence report from 18 cities conducted between April 17 and June 2, 2020 showed greatly varying levels of exposure in the general population during this period (Poustchi et al., 2020). This report also showed that 17.1% (95% CI 14.6–19.5%) of the population in these cities had been infected by the end of April 2020. The only systematic province-level study, which was conducted in Gilan during April 2020, showed that the adjusted prevalence of SARS-CoV-2 seropositivity was 22.2% (95% CI 16.4–28.5%) at the time (Shakiba et al., 2020). This is in agreement with our estimated exposure levels for Gilan based on the seasonal excess mortality trends in spring, i.e., 28% (95% CI 19–36%). Also, our provincial IFR estimates ranging from the lowest, 0.13%, in Sistan and Baluchistan to the highest, 0.36%, in Gilan are well-aligned with those of many other low-income countries (Brazeau et al., 2020).

In an earlier analysis (Ghafari et al., 2020b), the early transmission dynamics of the Iranian epidemic were reconstructed and it was projected that as the second peak emerged in the summer there would be a total of 9 million individuals (10.7% of the population) who had recovered by July 14, 2020. This is very close to our current estimate, 10.3%, for the nationwide exposure levels based on excess mortality data by June 20, 2020. Although we cannot independently verify the validity of any of the nationwide seroprevalence studies released to the press, they seem to be in general agreement with our estimates based on seasonal excess mortality data during the winter and spring. Assuming that the ratio between excess mortality and confirmed COVID-19-related deaths remains the same over time (i.e., approximately 2.5), by October 25, 2020 there would have been a total of approximately 84 000 COVID-19-related deaths (based on the 32 616 confirmed deaths at the time) and 33 million Iranians exposed to SARS-CoV-2, which is very close to the 35 million estimate based on the nationwide seroprevalence studies at the time (Hamshahri Online, 2020).

The present study also demonstrated that the mean excess mortality rate across the country had increased by more than 10-fold since the start of the epidemic and that all provinces had high mortality rates for at least one season as a consequence of the COVID-19 epidemic. In particular, most of the provinces that had a significant number of excess deaths in more than one season, experienced a surge in the death toll following the initial surge back in the winter and/or spring, which indicates that the epidemic was never fully brought under control. Given the record-high number of confirmed COVID-19-related deaths during the fall of 2020, we predict that the excess mortality counts will continue to increase across the country once NOCR updates the data for the fall of 1399 SH.

A previous epidemiological and phylogenetic analysis on individuals with a travel link to Iran showed that there were significant levels of under-reporting of cases and deaths, particularly in the early stages of the epidemic, which started back in mid- to late-January (Ghafari et al., 2020a). By comparing the mortality trends in provinces with a significant number of recorded deaths both in the fall (13 out of 31 provinces) and winter (five out of 31 provinces) of 2019–2020, it was determined that the unusually high mortality rates from September 23 to December 21, 2019 were not linked to any potential cryptic transmission of the virus across the country. More detailed investigations into the causes of the deaths and time-resolved monitoring for acute respiratory infections are required to determine the key source(s) of the spike during this period.

This study has some limitations. While COVID-19 has been directly responsible for the majority of excess deaths during the epidemic, it is possible that not all excess deaths were directly attributable to SARS-CoV-2 infection (Beaney et al., 2020, Viglione, 2020). We also note that the NOCR registration system might be strained when facing a potentially massive backlog of uncounted death certificates and prone to temporary under-counting. An earlier analysis showed that deaths due to other causes such as traffic injuries only fell by a few hundred when compared to previous years (Iranian Legal Medicine Organization, 2020) and this has not been a major contributor to the changing trend in excess mortality during the epidemic (Kadivar, 2020a, Kadivar, 2020b).

Given the limited number of data points per year, i.e., one data point for every season, the use of more complex models such as generalized linear models (GLMs) was avoided, which may demand more parameters to fit the data. In particular, the issue with using GLMs for our analysis would be that such models make no attempt to remove redundant parameters from the non-linear terms. As a result, fitting such models to only a few time points would risk overparameterization. However, GLMs are desirable for datasets with high temporal resolution (e.g., when excess mortality is recorded on a weekly or biweekly basis). Nevertheless, using a standard 5-year average to calculate the expected number of deaths can bias estimates of excess mortality, as this would not correctly account for significant changes in the excess mortality trend over time due to, for instance, a changing population size or other secular trends that are present in the NOCR data. It was also noted that although some provinces showed a seasonal trend in mortality with higher death rates during the fall and winter compared to the spring and summer, this pattern were not readily detectable for every province (i.e., variations during each season were high). Therefore, we also avoided using a periodic regression model, as this may have biased the estimates for expected seasonal deaths (Tadbiri et al., 2020). Such wide variations during each season also make it difficult to identify a baseline for the expected seasonal mortality in each province. To estimate the mean IFR for each province, we re-weighted the IFR of each age group by its respective share of the total population in each province. This procedure relies on the assumption that contagion was homogeneous between the age groups. This assumption was made because neither the all-cause mortality data from NOCR nor the reported daily cases and deaths from the MoHME provided any information about the age distribution of those with COVID-19. We also did not have any information about the social contact patterns across the country. Thus, this assumption may be partly violated due to reasons such as the heterogeneity in the burden of the epidemic in nursing homes where a subgroup of the population are interacting more frequently among themselves. However, as outlined in the Methods section, correction for such heterogeneities could be performed by adjusting the age-specific IFR estimates (O’Driscoll et al., 2020). In addition, a recent analysis showed that the increase in mortality by age from COVID-19 strongly resembles the age pattern of all-cause mortality (Goldstein and Lee, 2020). On the other hand, the potential age dependency of susceptibility to infection by SARS-CoV-2 may result in certain age groups becoming more prone to infection (Davies et al., 2020).

## Authors’ contributions

MG and AK designed the analysis. MG wrote the manuscript. MG and AKad conducted the analysis. All authors reviewed and edited the manuscript.

## Funding source

MG was funded by the Biotechnology and Biological Science Research Council (10.13039/501100000268BBSRC) grant number BB/M011224/1 and the Oxford-Radcliffe graduate scholarship from 10.13039/501100000734University College, Oxford.

## Ethical approval

None.

## Conflict of interest

We declare no conflicts of interest.

## References

[bib0005] Andersen K.G., Rambaut A., Lipkin W.I., Holmes E.C., Garry R.F. (2020). The proximal origin of SARS-CoV-2. Nat Med.

[bib0010] Beaney T., Clarke J.M., Jain V., Golestaneh A.K., Lyons G., Salman D. (2020). Excess mortality: the gold standard in measuring the impact of COVID-19 worldwide?. J R Soc Med.

[bib0015] Brazeau N., Verity R., Jenks S., Fu H., Whittaker C., Winskill P. (2020). Report 34: COVID-19 infection fatality ratio: estimates from seroprevalence.

[bib0020] Buss L.F., Prete C.A., Abrahim C.M.M., Mendrone A., Salomon T., de Almeida-Neto C. (2020). Three-quarters attack rate of SARS-CoV-2 in the Brazilian Amazon during a largely unmitigated epidemic. Science.

[bib0025] Checchi F., Roberts L., Humanitarian Policy Group (2005).

[bib0030] Davies N.G., Klepac P., Liu Y., Prem K., Jit M., Eggo R.M. (2020). Age-dependent effects in the transmission and control of COVID-19 epidemics. Nat Med.

[bib0035] Ghafari M., Hejazi B., Karshenas A., Dascalu S., Ferretti L., Ledda A. (2020). Ongoing outbreak of COVID-19 in Iran: challenges and signs of concern. medRxiv.

[bib0040] Ghafari M., Hejazi B., Karshenas A., Dascalu S., Ferretti L., Ledda A. (2020). Ongoing outbreak of COVID-19 in Iran: challenges and signs of concern with under-reporting of prevalence and deaths.

[bib0045] Ghafari M., Madani K. (2020). In search of the murder: making sense of Iran’s reported deaths. https://medium.com/@kavehmadani/in-search-of-the-murder-making-sense-of-irans-reported-deaths-4279d2b03175.

[bib0050] Goldstein J.R., Lee R.D. (2020). Demographic perspectives on the mortality of COVID-19 and other epidemics. Proc Natl Acad Sci U S A.

[bib0055] Hallal P.C., Hartwig F.P., Horta B.L., Silveira M.F., Struchiner C.J., Vidaletti L.P. (2020). SARS-CoV-2 antibody prevalence in Brazil: results from two successive nationwide serological household surveys. Lancet Global Health.

[bib0060] Iranian Legal Medicine Organization (2020). Motor vehicle crash statistics. https://www.lmo.ir/web_directory/53999-%D8%AA%D8%B5%D8%A7%D8%AF%D9%81%D8%A7%D8%AA.html.

[bib0065] Kadivar A. (2020). Corona’s footsteps in civil registration statistics [Persian]. https://blog.statsminute.ir/%d8%b1%d8%af-%d9%be%d8%a7%db%8c-%da%a9%d8%b1%d9%88%d9%86%d8%a7-%d8%af%d8%b1-%d8%a2%d9%85%d8%a7%d8%b1-%d8%ab%d8%a8%d8%aa-%d8%a7%d8%ad%d9%88%d8%a7%d9%84/.

[bib0070] Kadivar A. (2020). Increase in the number of registered deaths during spring [Persian]. https://blog.statsminute.ir/%D8%A7%D9%81%D8%B2%D8%A7%DB%8C%D8%B4-%D8%A7%D8%B3%D9%86%D8%A7%D8%AF-%D9%81%D9%88%D8%AA-%D8%AB%D8%A8%D8%AA-%D8%A7%D8%AD%D9%88%D8%A7%D9%84-%D8%AF%D8%B1-%D8%A8%D9%87%D8%A7%D8%B1-99/.

[bib0075] Khosravi A., Taylor R., Naghavi M., Lopez A.D. (2007). Mortality in the Islamic Republic of Iran, 1964–2004. Bull World Health Organ.

[bib0080] Khosravi A., Taylor R., Naghavi M., Lopez A.D. (2007). Differential mortality in Iran. Popul Health Metrics.

[bib0085] Lu J., du Plessis L., Liu Z., Hill V., Kang M., Lin H. (2020). Genomic epidemiology of SARS-CoV-2 in Guangdong Province, China. Cell.

[bib0090] National Committee on COVID-19 Epidemiology (2020). Summary of the scattered seroepidemiological reports from across the country [Persian].

[bib0095] (2020). New statistics on Coronavirus infections in Iran: 35 million people! [Persian]. https://www.hamshahrionline.ir/news/559881/%D8%A2%D9%85%D8%A7%D8%B1-%D8%AC%D8%AF%DB%8C%D8%AF-%D9%85%D8%A8%D8%AA%D9%84%D8%A7%DB%8C%D8%A7%D9%86-%DA%A9%D8%B1%D9%88%D9%86%D8%A7-%D8%AF%D8%B1-%D8%A7%DB%8C%D8%B1%D8%A7%D9%86-%DB%B3%DB%B5-%D9%85%DB%8C%D9%84%DB%8C%D9%88%D9%86-%D9%86%D9%81%D8%B1-%DB%B5%DB%B0-%D9%85%DB%8C%D9%84%DB%8C%D9%88%D9%86.

[bib0100] O’Driscoll M., Dos Santos G.R., Wang L., Cummings D.A.T., Azman A.S., Paireau J. (2020). Age-specific mortality and immunity patterns of SARS-CoV-2. Nature.

[bib0105] Poustchi H., Darvishian M., Mohammadi Z., Shayanrad A., Delavari A., Bahadorimonfared A. (2020). SARS-CoV-2 antibody seroprevalence in the general population and high-risk occupational groups across 18 cities in Iran: a population-based cross-sectional study. Lancet Infect Dis.

[bib0110] Rasambainarivo F., Rasoanomenjanahary A., Rabarison J.H., Ramiadantsoa T., Ratovoson R., Randremanana R. (2020). Monitoring for outbreak associated excess mortality in an African city: detection limits in Antananarivo, Madagascar. Int J Infect Dis.

[bib0115] Shakiba M., Nazari S.S.H., Mehrabian F., Rezvani S.M., Ghasempour Z., Heidarzadeh A. (2020). Seroprevalence of COVID-19 virus infection in Guilan province, Iran. Emerg Infect Dis.

[bib0120] Statistical Center of Iran (2020). Statistical Center of Iran. https://www.amar.org.ir/english.

[bib0125] Tadbiri H., Moradi-Lakeh M., Naghavi M. (2020). All-cause excess mortality and COVID-19-related deaths in Iran. Med J Islam Repub Iran.

[bib0130] United Nations Statistics Division (2015). Demographic and social statistics. https://unstats.un.org/unsd/demographic-social/crvs/.

[bib0135] Viglione G. (2020). The true toll of the pandemic. Nature.

[bib0140] Zeinalipour M. (2020). Serology tests; the basis for the report that 25 million Iranians have been infected to Corona [Persian]. https://www.isna.ir/news/99042921012/%D8%AA%D8%B3%D8%AA%D9%87%D8%A7%DB%8C-%D8%B3%D8%B1%D9%88%D9%84%D9%88%DA%98%DB%8C-%D9%85%D8%A8%D9%86%D8%A7%DB%8C-%D8%A2%D9%85%D8%A7%D8%B1-%DB%B2%DB%B5-%D9%85%DB%8C%D9%84%DB%8C%D9%88%D9%86%DB%8C-%D8%A7%D8%A8%D8%AA%D9%84%D8%A7%DB%8C-%D8%A7%DB%8C%D8%B1%D8%A7%D9%86%DB%8C%D8%A7%D9%86-%D8%A8%D9%87-%DA%A9%D8%B1%D9%88%D9%86%D8%A7.

